# Remnant Trees Affect Species Composition but Not Structure of Tropical Second-Growth Forest

**DOI:** 10.1371/journal.pone.0083284

**Published:** 2014-01-13

**Authors:** Manette E. Sandor, Robin L. Chazdon

**Affiliations:** Department of Ecology and Evolutionary Biology, University of Connecticut, Storrs, Connecticut, United States of America; University of Marburg, Germany

## Abstract

Remnant trees, spared from cutting when tropical forests are cleared for agriculture or grazing, act as nuclei of forest regeneration following field abandonment. Previous studies on remnant trees were primarily conducted in active pasture or old fields abandoned in the previous 2–3 years, and focused on structure and species richness of regenerating forest, but not species composition. Our study is among the first to investigate the effects of remnant trees on neighborhood forest structure, biodiversity, and species composition 20 years post-abandonment. We compared the woody vegetation around individual remnant trees to nearby plots without remnant trees in the same second-growth forests (“control plots”). Forest structure beneath remnant trees did not differ significantly from control plots. Species richness and species diversity were significantly higher around remnant trees. The species composition around remnant trees differed significantly from control plots and more closely resembled the species composition of nearby old-growth forest. The proportion of old-growth specialists and generalists around remnant trees was significantly greater than in control plots. Although previous studies show that remnant trees may initially accelerate secondary forest growth, we found no evidence that they locally affect stem density, basal area, and seedling density at later stages of regrowth. Remnant trees do, however, have a clear effect on the species diversity, composition, and ecological groups of the surrounding woody vegetation, even after 20 years of forest regeneration. To accelerate the return of diversity and old-growth forest species into regrowing forest on abandoned land, landowners should be encouraged to retain remnant trees in agricultural or pastoral fields.

## Introduction

Second-growth tropical forests provide a new source of hope for biodiversity conservation in an era of rapid destruction of old-growth tropical forests [1], [2], [3]. Second-growth vegetation colonizing abandoned agricultural settings encounters many challenges, including seed dispersal limitation, competition with grasses or ferns, and harsh environmental conditions for establishment [4], [5], [6]. Remnant trees—old-growth forest trees left standing on anthropogenically modified land—can potentially accelerate forest regeneration following pasture abandonment by facilitating seed dispersal into the pasture. This facilitation by remnant trees can occur by attracting frugivorous birds and bats, serving as seed sources, and ameliorating abiotic conditions beneath their crowns (i.e. [7], [8], [9]). Remnant trees are ubiquitous across the tropics, and have been shown to serve as “regeneration nuclei” for secondary forest regrowth in many tropical regions [7], [10], [11]. Current theories about how remnant trees function as regeneration nuclei are largely based on studies conducted in active pasture or within a few years after pasture abandonment ([12], but see [13]). Further, most of these studies investigate effects of remnant trees on the structure and species richness of regenerating forest, but do not evaluate effects on composition of species (i.e. [14], [15], but see [13]).

During the initial stages of regeneration, remnant or isolated pasture trees significantly impact regeneration through enhanced seed dispersal, seed germination, and seedling growth. Seeds are deposited in greater numbers, with greater species richness, and with a greater abundance of animal-dispersed species under remnant trees than away from the edge of the crown [6], [15], [16]. Changes in levels of irradiance, air temperature, and soil moisture beneath crowns of remnant trees may affect seed germination and seedling growth, once seeds arrive [8]. Regenerating vegetation has a higher density, greater basal area, and higher species richness under remnant trees than in plots away from a remnant tree [6], [14], [17]. Schlawin and Zahawi (2008) found that regeneration near remnant trees 20 years after pasture abandonment had higher basal area, stem density, and species richness than areas away from remnant trees [13]. Until now, this was the only study to address the effects of remnant trees on surrounding vegetation a few decades after forest regeneration, long after canopy closure and stand initiation.

Our study is among the first to investigate effects of remnant trees on the structure and composition of woody regeneration in 20-year old stands of second-growth forest. Studying the effects of remnant trees in older second-growth forest allows us to make inferences about the longer-term effects of remnant trees on regrowth of the surrounding woody vegetation. Understanding the effects of remnant trees on forest regrowth can also enhance management by helping determine the benefits of sparing individual trees when clearing forest. We compare forest regeneration in sample plots surrounding remnant trees to plots lacking remnant trees within the same forest stand. For stems ≥1 cm diameter at breast height (DBH) we compare measures of forest structure between plot types. For stems ≥5 cm DBH we compare measures of species diversity, species composition, abundance of individuals by dispersal mode, and successional specialist groups between plot types. We also compare the composition of sample plots with similar sample areas in forest monitoring plots in nearby second-growth (5–50 yr old) and old-growth forests.

## Methods

### Ethics Statement

Our study took place on protected land owned by the nonprofit organization Osa Conservation and the private nature reserve Lapa Ríos Ecolodge and Wildlife Reserve. We obtained permission from both organizations to access their land. Voucher specimens were collected under collecting permit Resolución No. 069-2011-SINAC, Sistema Nacional de Áreas de Conservación. Voucher specimens were archived with Reinaldo Aguilar on the Osa Peninsula. *Caryocar costaricense* J.D. Sm. is protected under CITES Appendix II. *Cedrela odorata* L. is protected under CITES Appendix III. Data are available on Harvard Dataverse Network (http://thedata.harvard.edu/dvn/dv/MESandor).

### Site Description

We conducted our study in lowland tropical humid forest [18] on the Osa Peninsula, Puntarenas, Costa Rica. The flora and fauna of the Osa Peninsula are diverse with a high level of endemism [19], [20], [21], [22], yet few published studies have been conducted there. The mean annual temperature on the Peninsula is 24–28°C, and rainfall is 4.0–5.0 m/yr [23], [24], [25]. The wet season is from May to November, with a peak in September and October, and the dry season is January to April. The area receives less than 10 mm of precipitation in a month during the dry season with the exception of April [25]. Upper elevations are generally cooler (by 3–4°C) and receive less rainfall (<1 m/yr difference) than lowland areas [25].

Our study sites were in second-growth forests on land owned by Osa Conservation (OC, 8.41°N, 83.34°W) and Lapa Ríos Ecolodge and Wildlife Reserve (LR, 8.39°N, 83.30°W) (Figure S1). The OC study site is within a floodplain, with an elevational range of 37–76 m above sea level. The LR study site consists of rolling hills, with an elevational range of 233–283 m above sea level. Soils are primarily ultisols at both sites [26], [27].

The second-growth forest sites are former cattle pastures. At the time of study, the second-growth forest was 20 years old (at LR) or 23 years old (at OC) with a historical pasture use of between 20 and 30 years (at LR) or about 20 years (at OC). We verified land use history from conversations with Osa Conservation staff, interviews with local landowners, and aerial photographs obtained from the Instituto Geográfico Nacional (San Jose, Costa Rica). The forest at both sites has closed canopies (15–30 m high, depending on site and forest patch). Areas surrounding the study locations are primarily old growth or second-growth forest, with a few large cleared tracts of pasture remaining to the west of the OC site and to the south of the LR site (Figure S1).

### Study Design

We conducted all surveys between May and July, 2011. We located 10 remnant trees at least 50 m from the nearest old-growth forest edge, with no other large (>75 cm DBH) trees within 30 m of the remnant tree (Methods S1 in File S1, Figure S1). To confirm remnant tree status we matched GPS points of the remnant trees to isolated trees in pasture observed in historical aerial photographs from the Instituto Geográfico Nacional from 1976, 1980, 1992, and 1995. We also located 20 typical (control) trees in nearby areas of second-growth forest lacking remnant trees. The central trees of control plots were generally located 50–150 m away from the remnant trees, had no trees >75 cm DBH within 30 m, and were >50 m away from the nearest old-growth forest edge (Figure S1, Table S1 in File S1). Remnant trees were generally considerably taller and larger than central trees in control plots (Table S1 in File S1) and all other trees in the study. Remnant trees represented species generally found in old-growth forest and were distinct species from central trees in the control plots, which comprised species commonly found in second-growth forest (Table S1 in File S1). The 10 remnant trees represented 9 species and 8 families (Table S1 in File S1).

We placed four 5 m wide by 30 m long survey transects at 90-degree angles to each other around the center tree (either a remnant or a “control tree”). Each transect consisted of six 5 m by 5 m quadrats (total of 24 quadrats per plot). We identified all measured stems ≥5 cm DBH to species or morphospecies in the field, with the exception of understory trees in the genus *Piper*, which were identified to genus (5.14% of all identified stems). Reinaldo Aguilar, the regional botanical expert, collected and identified voucher specimens for individuals we could not identify in the field. We measured the DBH of all non-liana woody stems ≥1 cm DBH in each transect, but we did not identify stems ≥1 cm and <5 cm DBH. We counted canopy and understory trees with multiple stems as one individual. In a stratified subsample of 16 remnant and control plots (6 remnant tree plots, 10 control plots), we counted all free-standing, woody seedlings >30 cm in height and <1 cm DBH in 1 m by 1 m square quadrats at 2.5 m and 7.5 m from the central tree along each of the four sampling transects.

### Vegetation Structure and Diversity

We calculated tree density (≥1 cm DBH), basal area (≥1 cm DBH), seedling density (<1 cm DBH), extrapolated species richness (≥5 cm DBH), species diversity (≥5 cm DBH), and species evenness (≥5 cm DBH) for the regenerating vegetation in each remnant or control plot (all quadrats surveyed around a central tree). We excluded any quadrats with geographic barriers to regeneration, such as streams or trails, from all calculations and analyses (≤4 quadrats excluded within a single plot for a total survey area of 500–600 m^2^ per plot). To compare species richness between remnant and control plots, we created species accumulation curves using EstimateS (version 9), extrapolated to the greatest number of individuals in a single plot [28], [29]. We estimated local species diversity per plot as Shannon exponential diversity for stems ≥5 cm DBH, using EstimateS [28]. Because some plots had fewer quadrats than others, we used Shannon exponential diversity at the greatest common number of quadrats for all plots. We calculated species evenness as Shannon evenness (Shannon diversity divided by the natural log of richness) for all stems ≥5 cm DBH.

### Statistical Analyses

To assess whether or not the presence of a remnant tree affected basal area, tree density, seedling density, species richness, species diversity, or species evenness, we conducted two-way ANOVAs in R, using presence or absence of a remnant tree and site (OC or LR) as fixed effects (to correct for any site effects) [30]. We assessed all pairwise similarities for species composition between remnant and control plots [28], [31] using the Chao-Jaccard abundance-based estimator of similarity [32]. We calculated similarity between the second-growth woody vegetation in remnant and control plots (≥5 cm DBH) with woody vegetation in nearby old-growth forests, using data from nearby forest survey plots (≥5 cm DBH) [27]. Because the nearby forest survey plots (0.5 ha) were much larger than the remnant or control plots (500–600 m^2^), we separated them into 10 discrete 500 m^2^ subplots, each comprising five 10 m^2^ quadrats. These quadrats were arranged in a linear configuration, unlike the cross configuration of our remnant and control plots. We calculated pairwise similarities using all of these subplots and then averaged the results between a single remnant or control plot and all the subplots.

To assess whether or not the presence of a remnant tree affected pairwise similarity to old-growth forest, we conducted two-way ANOVAs, using presence or absence of a remnant tree and site as fixed effects. We performed a linear regression analysis on the mean pairwise similarities between second-growth plots (remnant and controls) and nearby old-growth forest to determine if proximity to old-growth forest was correlated with pairwise similarity. All remnant trees and central trees within control plots were within 315 m of the edge of old-growth forest (Figure S1). We used analysis of similarities (ANOSIM), to test whether or not the differences in species composition between forest composition of remnant plots and control plots were statistically significant [31].

Based on a multinomial statistical method for classifying habitat specialists and generalists [33], we classified 278 tree species ≥5 cm DBH within the nearby eleven 0.5 ha forest survey chronosequence plots [27] into old-growth specialists (“OG Specialist”), second-growth specialists (“SG Specialist”), generalists (“Generalist”), and too rare to classify (“Rare”). We conducted the analysis using CLAM [34] with a *p*-level of 0.001 and a *k*-level of 0.667 (both highly conservative). We then applied these classifications to the species sampled in remnant and control plots. We used an analysis of multinomial proportions with uninformative priors to determine the proportion of each category of trees in remnant and control plots using R2OpenBUGS in R [35], [36], [37]. We did not include *Piper* spp. in similarity or specialist classification analyses because they were identified only to genus. We also excluded morphospecies from the specialist classification analyses.

### Dispersal Mechanisms

We determined dispersal vectors (wind, explosive, animal, gravity) for all woody species recorded in the study from information found in the primary literature. We used an analysis of multinomial proportions with uninformative priors to compare the proportion of each dispersal mechanism in remnant and control plots using R2OpenBUGS in R [35], [36], [37].

## Results

### Local richness and diversity of the regenerating forest

**Figure 1:**
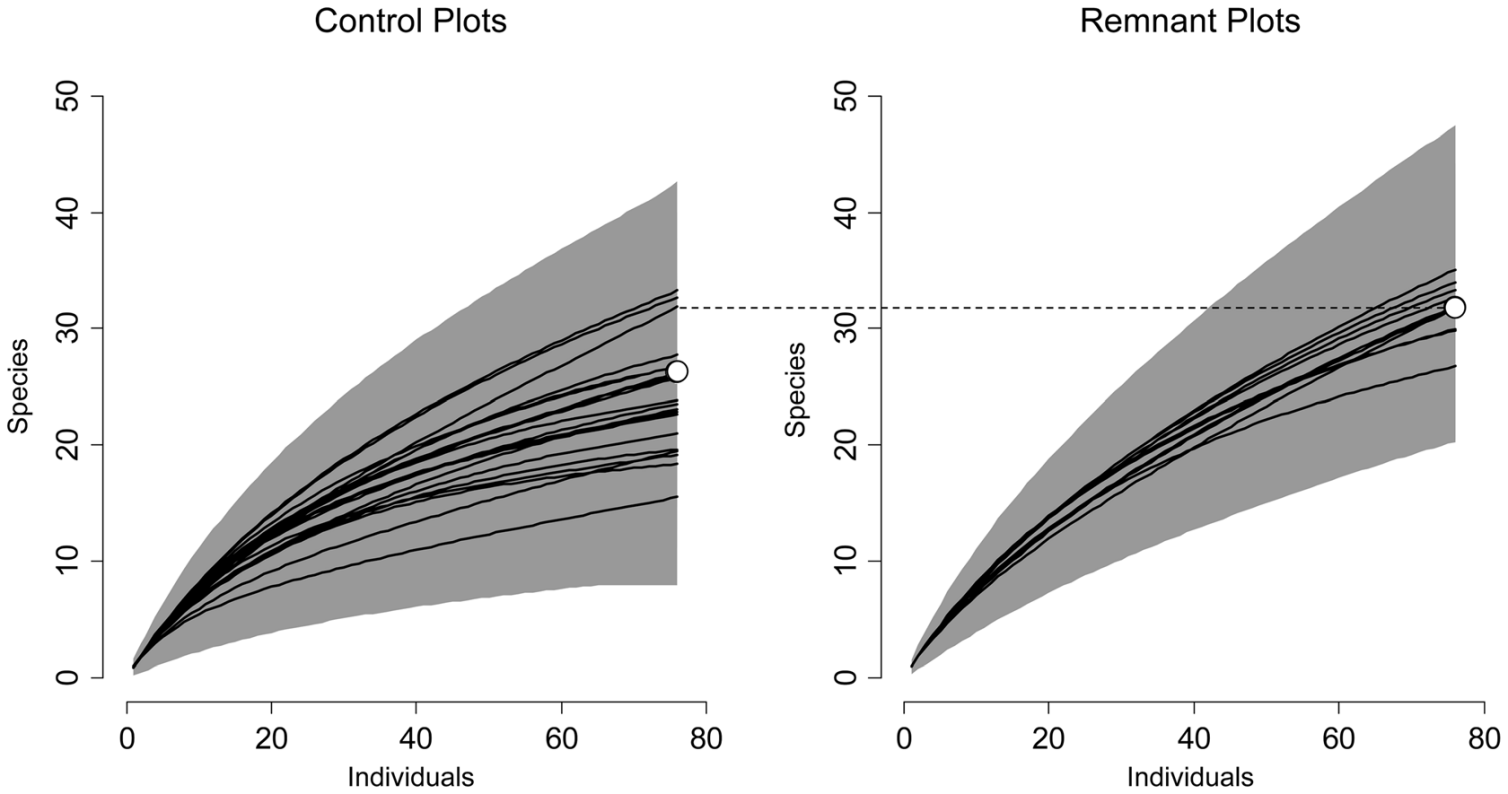
Extrapolated species accumulation curves. Species accumulation curves for remnant and control plots, for all stems ≥5 cm DBH. All species accumulation curves are extrapolated to 76 individuals, the greatest number of individuals found in one plot (min individuals in a plot  = 30, mean  = 53.6). White dots indicate mean species richness for all remnant or control plots. The dashed line allows for comparison of remnant and control plot means.

We found a total of 171 species in the surveyed quadrats representing 49 families, composed of 117 species representing 42 families in the 10 remnant tree plots (227 quadrats, each 25 m^2^, all stems ≥5 cm DBH) and 131 species representing 42 families in the 20 control plots (461 quadrats, each 25 m^2^, all stems ≥5 cm DBH). Extrapolated species richness for all stems ≥5 cm DBH was significantly greater in the remnant tree plots than in the control plots by an average of 7 species (Table 1, Figure 1). Remnant tree plots also showed significantly higher species diversity for stems ≥5 cm DBH (measured by Shannon exponential diversity) than control plots (Table 1). Evenness did not differ significantly between remnant and control plots for stems ≥5 cm DBH (Table 1).

**Table 1:** Effect of remnant tree or site on species structure and diversity.

	Presence of remnant tree		Site	
	F	p	F	p
Basal area ≥1 cm DBH	0.18	0.673	0.79	0.382
Seeding (>1 cm DBH) density	0.01	0.942	0.23	0.641
**Density ≥1 cm DBH**	2.74	0.110	**11.06**	**0.003**
Light	1.73	0.211	0.00	0.998
**Extrapolated species richness ≥5 cm DBH**	**20.96**	**<<0.001**	1.24	0.275
**Species diversity ≥5 cm DBH**	**5.83**	**0.023**	1.75	0.196
Species evenness ≥5 cm DBH	0.07	0.794	0.10	0.757
**Pairwise similarity (trees to old-growth forest)**	**27.62**	**<<0.001**	**12.19**	**0.002**

ANOVA results are shown for basal area, seedling density, tree density, light, species richness, species diversity, species evenness, and pairwise similarity to old-growth forest (df  = 1 and residual df  = 28 for all). Results with significant p-values are shown in bold.

### Species composition

We found 73 species shared between remnant and control plots, 41 species only in remnant tree plots, and 56 species only in control plots. Abundances of the top-ranked species in remnant and control plots were similar, with neither displaying more dominance than the other (Table S3 in File S1). A few species showed a higher relative abundance (>2% difference) around remnant trees than in control plots: *Socratea exorrhiza* (Mart.) H. Wendl., *Chimarrhis latifolia* Standl., *Tetrathylacium macrophyllum* Poepp., *Croton schiedeanus* Schltdl., and *Alchornea costaricensis* Pax & K. Hoffm. (Table S3 in File S1). In contrast, *Apeiba tibourbou* Aubl., *Luehea seemannii* Triana & Planch., *Lacistema aggregatum* (P.J. Bergius) Rusby, *Spondias mombin* L., *Cordia bicolor* A. DC., *Simaba cedron* Planch., and *Casearia sylvestris* Sw. showed a much higher abundance (>2% difference) in control plots than around remnant trees (Table S3 in File S1). Trees found in one type of plot (remnant or control) but not in the other were generally at low relative abundances in the plot type in which they were found (<1% relative abundance). Three species were exceptions: *Casearia sylvestris* Sw., *Psychotria grandis* Sw., and *Palicourea guianensis* Aubl. were all found in control plots at relative abundances of >1% and <2.3%. Species composition in remnant and control plots differed significantly for all stems ≥5 cm DBH (ANOSIM; R = 0.161, p = 0.021). Including *Piper* spp. in the analysis did not affect the significance of this result (R = 0.150, p = 0.044).

Old-growth forest plots showed significantly higher compositional pairwise similarity to remnant tree plots than to control plots (Table 1). Pairwise similarity was not correlated with distance to nearest old-growth forest (all plots: R^2^ = 0.053, p = 0.220), even when grouped by remnant or control plots (for control plots: R^2^ = 0.019, p = 0.563; for remnant tree plots: R^2^ = 0.007, p = 0.822).

### Generalists and Specialists

Most species in our survey were present in the chronosequence survey. Ten OG Specialist species, 20 SG Specialist species, and 18 Generalist species were recorded in our sample plots (Table S4 in File S1). Thirty-eight of the 170 species found in our study were not found in the chronosequence survey [27] and therefore were not classified in the analysis; we classified these species as Rare (Table S4 in File S1).

**Figure 2:**
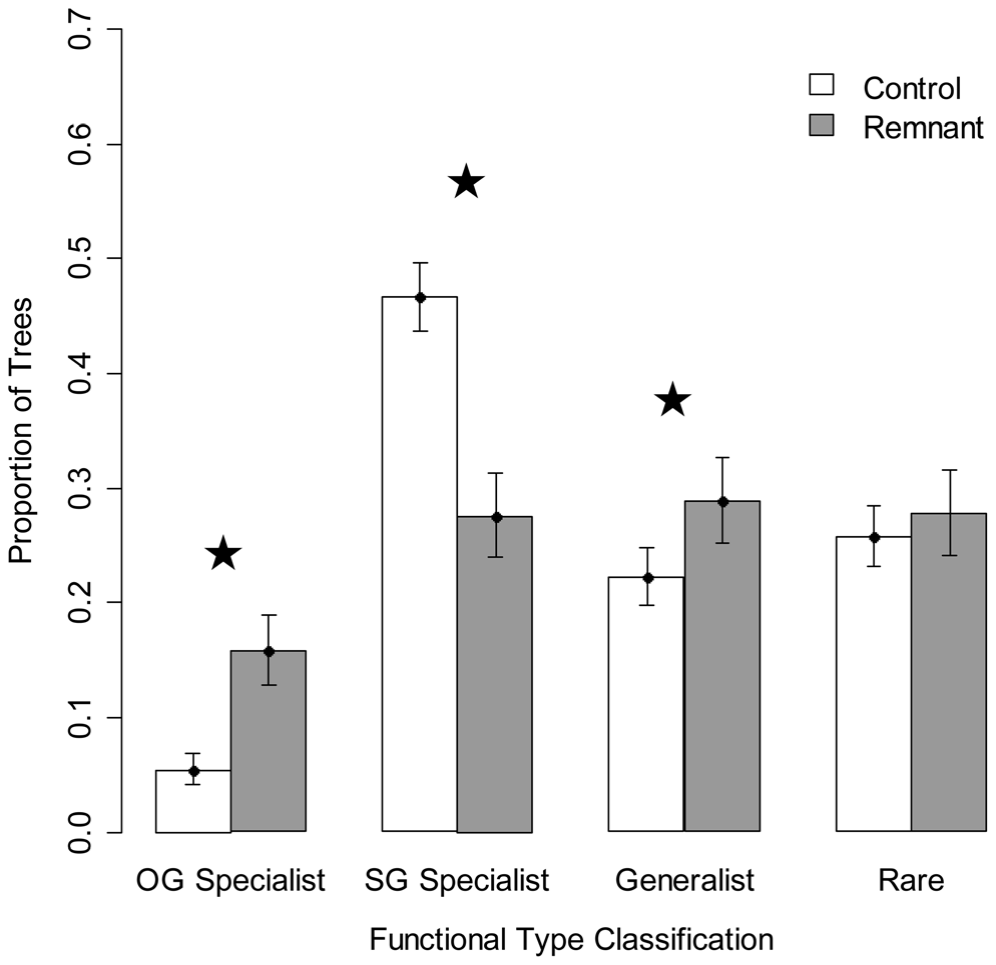
Relative proportions of specialists in control and remnant tree plots. Relative proportions of each classification type found in control and remnant tree plots for all stems ≥5 cm DBH. Proportions are such that all four categories for one focal tree type sum to 1. Statistical significance was assessed by whether or not the Bayesian posterior 95% confidence intervals of the proportions overlapped between control and remnant plots, and it is indicated with a star.

For stems ≥5 cm DBH, we observed significantly larger proportions of OG Specialist and Generalist trees in remnant tree plots than in control plots (by 10.4±4.4% and 6.7±6.3%, respectively; Figure 2). We also observed a significantly larger proportion of SG Specialist trees in control plots compared to remnant tree plots (by 19.1±6.8%; Figure 2). Similar trends emerged when we partitioned the data by site. None of the OG Specialist or Generalist species found around remnant trees were conspecifics. Only one remnant tree, *Coccoloba tuerckheimii* Donn. Sm. had a conspecific tree (≥5 cm DBH) in the surrounding sampled quadrats. This species was classified as Rare.

To determine whether the significant differences in OG Specialists and SG Specialists between remnant and control plots were due to abundance, we reduced the ≥5 cm DBH abundance data to incidence data. Control plots had a few SG Specialist species that were not found around remnants (*Miconia argentea* (Sw.) DC., *Palicourea guianensis* Aubl., *Trichospermum galeottii* (Turcz.) Kosterm.), and the remnant tree plots had a few OG Specialist species that were not found in control plots (*Cheiloclinium cognatum* (Miers) A.C. Sm., *Guarea kunthiana A. Juss.*, *Gustavia brachycarpa* Pittier, *Pseudolmedia spuria* (Sw.) Griseb.). Despite these species differences, we found no significant differences in the proportion of species of OG Specialists or SG Specialists in remnant vs. control plots, confirming that the presence of a remnant tree affected abundance rather than incidence of these ecological groups.

### Dispersal Mechanisms

Nearly 88% of the species (82.5% of individuals) found in the plots were animal-dispersed, 10.3% of species (14.3% of individuals) were wind-dispersed, and 1.7% of species (3.2% of individuals) used other dispersal mechanisms such as explosion and gravity. Percentage of animal-dispersed individuals within a plot ranged from 51.5 to 100%. Percentage of wind-dispersed individuals within a plot ranged from 0 to 39.2%. We found no significant differences in the proportion of animal-dispersed individuals or wind-dispersed individuals in remnant tree plots, compared with the proportion in control plots.

### Structure of the regenerating forest

Despite the striking differences in species composition, remnant tree plots and control plots did not differ significantly in terms of vegetation structure. Basal area of remnant and control plots did not differ significantly for stems ≥1 cm DBH (Table 1). Seedling (<1 cm DBH) density did not differ significantly between quadrats in remnant and control plots (Table 1). The presence of a remnant tree did not significantly affect the density of surrounding woody vegetation for stems ≥1 cm DBH (Table 1).

## Discussion

Our study is the first to demonstrate that remnant trees have a lasting effect on the species composition of the surrounding regenerating forest 20 years after land abandonment, while no longer having a detectable effect on vegetation structure. The presence of a remnant tree significantly affected all measures of species composition used in our study, with the exception of species evenness (Table 1). We observed higher extrapolated species richness and species diversity in second-growth forest plots with remnant trees compared to plots in the same stand lacking remnant trees (Table 1). Species richness and diversity results are congruent with other studies of remnant trees, including that by Schlawin and Zahawi (2008) in 23 year-old regenerating forest [6], [13], [17]. Furthermore, isolated remnant trees enriched surrounding tree assemblages with old-growth and generalist species, increasing the similarity of species composition to old-growth forest (Table 1, Figure 2). No other study has compared specialist group abundance between remnant trees and control plots, nor have they compared species composition around remnant trees with that of old-growth forest. Studies that focus on remnant trees in later stages of forest growth, beyond 30 years after agricultural abandonment, are needed to indicate whether or not this effect of remnant trees on many aspects of species composition continues.

We did not find any significant differences in forest structure between remnant and control plots, which is the opposite of what we expected based on previous studies, including that by Schlawin and Zahawi [13], [14], [17]. Our second-growth forest sites had closed canopies of strongly uniform heights (M. Sandor, pers. obs.) with similar understory light measurements in all surveyed plots (Table 1, Table S2 in File S1), unlike in any previous study [13], [14], [17]. This evidence suggests that regeneration of the 20 year old second-growth forests was sufficiently advanced that the forest structure had converged, erasing any potential earlier signal of enhanced regeneration or altered microclimates in the forest around our remnant trees. If our second-growth forest sites were beyond stand initiation phase and had progressed to stem exclusion or understory re-initiation [38], advanced forest structure in remnant tree plots would no longer be expected to have greater tree density, more seedlings, or higher basal area.

Even after 20 years of forest regrowth, remnant trees may enrich forest regeneration, but separating legacy effects from continuing effects is difficult. Generally speaking, smaller size classes of trees are younger, more recently established vegetation (5–9.9 cm DBH), and larger size classes are trees that established earlier in succession (≥10 cm DBH). Taking out the smaller size classes resulted in a lack of significant differences in extrapolated species richness and species diversity (Methods S1 in File S1, Results S1 in File S1), indicating that the species composition in remnant tree plots may not simply be a legacy effect from earlier stages of regeneration. Rather, remnant trees appear to continue to enhance the richness and diversity of species in the surrounding woody vegetation during later stages of forest succession.

Remnant trees can affect regeneration in three ways: by altering abiotic conditions under their crowns, by serving as seed sources for regenerating forest, and by attracting animal dispersers (i.e. [7], [8], [9]). Although light and other abiotic conditions likely would have been different early in the regeneration process, we found no differences in light conditions between remnant and control plots, suggesting that tree species were not responding to that particular abiotic condition at this stage of regeneration (Table 1, Table S2 in File S1). We only found one individual whose species matched that of the focal remnant tree. Further, we only found a few presumed offspring of the central remnant trees in the 1.0–4.9 cm DBH size class per remnant tree (M. Sandor, pers. obs.), indicating that the offspring of the remnant trees contribute little to the species composition found around these trees. The proportion of animal-dispersed individuals around remnant trees was not significantly different than in control plots, which we would not expect if animal-mediated seed dispersal was playing an important role in driving the differences in species composition between remnant trees and control plots. This leaves us without an explanation based on the factors we measured for what is driving the differences in composition around remnant trees in our study area. Remnant trees could be attracting a different suite of animal species, such as ones from nearby old growth forest, that are bringing more species of seeds and more OG Specialist seeds.

Like remnant trees, early colonizing, rapidly growing trees may also play an important role in enhancing local regeneration by attracting seed-dispersing frugivores in later stages of succession. Other studies have used the term “isolated trees” in pasture to show similar effects as those observed with remnant trees (e.g. [6], [16], [39], [40], [41], [42]). Focusing on isolated trees allows inclusion of trees that recruit after the forest is cleared for pasture (for example, trees with seedlings avoided by cattle), trees that are planted by the landholder for personal use (like fruit, fodder, or timber trees), and living fences [43]. Five of the control plots in our study had a presumed isolated tree at the center that was larger than most of the surrounding second-growth forest vegetation, but generally smaller than a remnant tree. If these trees affect the surrounding woody vegetation in ways similar to remnant trees, our results may not reflect the full magnitude of effects that would be seen if our control plots contained no isolated trees as the central tree. Studies that consider the differences–and similarities–in function between isolated trees and remnant trees could inform our understanding of succession through nucleation [10] as well as ecological reforestation methods that use native tree plantings in former agricultural land (“assisted regeneration”) [2].

Landscape effects play a large role in forest regeneration [44]. Greater proportions of old-growth forest in the landscape increase the regeneration capacity of abandoned pastures [45], [46]. Proximity to old-growth forest could also positively affect regeneration around a remnant tree or in open pasture, although neither our study nor Laborde *et al*. (2008) found any significant relationship [47]. In our study, old-growth forest was never more than 315 m away and in most cases was closer than 200 m away (Table S1 in File S1). The restricted range of distances from old-growth forest in our study could explain the lack of relationship between proximity to old-growth forest and level of regeneration. Further, the large percent of old-growth forest in the overall landscape of our study might have contributed to the accelerated regeneration of our sites compared with those used by Schlawin and Zahawi (Figure S1); our results may have been very different if our study had been conducted in a landscape largely devoid of nearby old-growth forest [13].

Remnant trees can enhance seed dispersal by providing “stepping stones” in the landscape for volant seed dispersers [40], [48], [49]. Remnant trees can occur in clusters or patches (tree “islands”) [50]. Seeds dispersed under remnant trees may come largely from other pasture trees or from these tree islands rather than from nearby forest [6], [16], [40], indicating that the abundance of other remnants, isolated trees, or tree islands within the vicinity may also affect regeneration around a particular remnant. Both the effects of proximity to old-growth forest and of nearby remnant or isolated trees or tree islands on the regeneration of forest, argue for a need to incorporate landscape factors into future studies and management of forest regeneration.

Second-growth forests, whether or not remnant trees are present, can be important for species conservation by fostering the establishment and growth of rare species. In our survey, 38 out of the 162 tree species (nearly 25% of all species) we recorded in remnant and control plots were not present in the eleven 0.5 ha chronosequence plots nearby. All of these species were uncommon, with fewer than 7 individuals found in all of our quadrats. While the importance of conserving old-growth forests is indisputable, second-growth forests represent a reservoir of an additional set of rare species worth conserving [3], [27]. When present, remnant and isolated pasture trees can be integral components of forest restoration and conservation strategies. Remnant trees enhance woody plant species biodiversity of the neighboring forest and could be used as indicators of higher biodiversity where they are present, as our study shows. They may help conserve animal diversity by providing an additional source of food in the landscape matrix or by providing breeding sites for native birds [51], [52] or roosts for bat species [53]. They also may enhance tree gene flow across the landscape and therefore enhance genetic diversity [54]. Because of these conservation benefits, their contribution to landscape-scale seed dispersal, and the enhanced forest regeneration shown in our study and other studies, landowners in the tropics should be encouraged to leave some trees standing when forest is converted to agricultural uses.

## Supporting Information

File S1 **Supporting Information Document.** Resumen, Methods S1, Results S1, and Tables S1-4.
(DOCX)

Figure S1 **Land cover change and tree plot locations at Osa Conservation and Lapa Ríos.** Remnant trees (white dots), and control trees (central trees in control plots; white triangles) on A) and C) Osa Conservation land and B) and D) Lapa Ríos Ecolodge and Wildlife Reserve land in A) and B) 1976 and C) and D) 2013. The white lines on the 1976 aerial photographs indicate the boundary of old-growth forest at greatest known level of clearing (not necessarily 1976). The images were processed in ArcMap 10.1. Photographs from 1976 are courtesy of Instituto Geográfico Nacional (San Jose, Costa Rica). Current (2013) satellite imagery is from ESRI basemap World_Imagery layer; satellite imagery sources are cited in the bottom right of both of the images.
(TIF)

## References

[1] Wright SJ (2010) The future of tropical forests. Yr Ecol Conserv Biol 2010 1195: 1–27. 10.1111/j.1749-6632.2010.05455.x 20536814

[2] Lamb D, Erskine P, Parrotta J (2005) Restoration of degraded tropical forest landscapes. Science 310: 1628–32. 16339437 10.1126/science.1111773

[3] Chazdon RL, Peres CA, Dent D, Sheil D, Lugo AE, et al (2009) The potential for species conservation in tropical secondary forests. Conserv Biol 23: 1406–17. 20078641 10.1111/j.1523-1739.2009.01338.x

[4] Nepstad DC, Uhl C, Pereira CA, Cardoso da Silva JM (1996) A comparative study of tree establishment in abandoned and mature forest of eastern Amazonia. Oikos 76: 25–39.

[5] Cubina A, Aide T (2001) The effect of distance from forest edge on seed rain and soil seed bank in a tropical pasture. Biotropica 33: 260–7.

[6] Slocum M (2001) How tree species differ as recruitment foci in a tropical pasture. Ecology 82: 2547–59.

[7] Guevara S, Purata S, VanDerMaarel E (1986) The role of remnant forest trees in tropical secondary succession. Vegetatio 66: 77–84.

[8] Manning AD, Fischer J, Lindenmayer DB (2006) Scattered trees are keystone structures - implications for conservation. Biol Conserv 132: 311–21.

[9] Nadkarni NM, Haber WA (2009) Canopy seed banks as time capsules of biodiversity in pasture-remnant tree crowns. Conserv Biol 23: 1117–26. 19438870 10.1111/j.1523-1739.2009.01235.x

[10] Yarranton GA, Morrison RG (1974) Spatial dynamics of a primary succession: Nucleation. J Ecol 62: 417–28.

[11] McDonnell M, Stiles E (1983) The structural complexity of old field vegetation and the recruitment of bird-dispersed plant-species. Oecologia 56: 109–16. 28310777 10.1007/BF00378225

[12] Murray KG, Winnett-Murray K, Roberts J, Horjus K, Haber WA, et al.. (2008) The roles of disperser behavior and physical habitat structure in regeneration of post-agricultural fields. In: Myster, RW, editor. Post-agricultural succession in the Neotropics. New York: Springer Science+Business Media, LLC. p. 192–215.

[13] Schlawin J, Zahawi RA (2008) “Nucleating” succession in recovering Neotropical wet forests: The legacy of remnant trees. J Veg Sci 19: 485–92.

[14] Carriere S, Letourmy P, McKey D (2002a) Effects of remnant trees in fallows on diversity and structure of forest regrowth in a slash-and-burn agricultural system in southern Cameroon. J Trop Ecol 18: 375–96.

[15] Carriere S, Andre M, Letourmy P, Olivier I, McKey D (2002b) Seed rain beneath remnant trees in a slash-and-burn agricultural system in southern Cameroon. J Trop Ecol 18: 353–74.

[16] Slocum M, Horvitz C (2000) Seed arrival under different genera of trees in a Neotropical pasture. Plant Ecol 149: 51–62.

[17] Elmqvist T, Wall M, Berggren A, Blix L, Fritioff A, et al (2002) Tropical forest reorganization after cyclone and fire disturbance in Samoa: Remnant trees as biological legacies. Conserv Ecol 5: 10.

[18] Holdridge LR, Grenke WC, Hatheway WH, Liang T, Tosi JA (1971) Forest Environments in Tropical Life Zones: A Pilot Study. New York: Pergamon Press.

[19] Janzen DH (1983) Costa Rican Natural History. Edit. Chicago: The University of Chicago Press. p. 619.

[20] Quesada Quesada FJ, Jiménez Madrigal Q, Zamora Villalobos N, Aguilar Fernández R, González Ramirez J (1997) Arboles de la península de Osa. Heredia, Costa Rica: INBio.

[21] Barrantes G, Jiménez Q, Lobo J, Maldonado T, Quesada M, et al.. (1999) Manejo forestal y realidad nacional en la península de Osa. San José, Costa Rica: INBio.

[22] Aguilar R, Cornejo X, Bainbridge C, Tulig M, Mori SA (2008 onward) Vascular Plants of the Osa Peninsula, Costa Rica. The New York Botanical Garden, Bronx, New York. Available: http:sweetgum.nybg.org/osa/.

[23] Herrera W (1986) Clima de Costa Rica. Edit. San José, Costa Rica: Universidad Estatal a Distancia. p. 118.

[24] Kernan C, Fowler N (1995) Differential substrate use by epiphytes in Corcovado National Park, Costa Rica a source of guild structure. J Ecol 83: 65–73.

[25] Hijmans RJ, Cameron SE, Parra JL, Jones PG, Jarvis A (2005) Very high resolution interpolated climate surfaces for global land areas. Int J Climatol 25: 1965–1978.

[26] Zambrano A MA, Broadbent EN, Durham WH (2010) Social and environmental effects of ecotourism in the Osa Peninsula of Costa Rica: the Lapa Rios case. J Ecotourism 9: 62–83.

[27] Morales Salazar MS, Vílchez Alvarado B, Chazdon R, Ortiz Malavasi E, Guevara Bonilla M (2013) Estructura, composición y diversidad vegetal en bosques tropicales del Corredor Biológico Osa, Costa Rica. Revista Forestal Mesoamericana Kurú 10: 1–13.

[28] Colwell RK (2012) EstimateS: statistical estimation of species richness and shared species from samples. Version 9. User's Guide and application. Available: http://purl.oclc.org/estimates.

[29] Colwell RK, Chao A, Gotelli NJ, Lin S, Mao CX, et al (2012) Models and estimators linking individual-based and sample-based rarefaction, extrapolation and comparison of assemblages. J of Plant Ecol 5: 3–21.

[30] R Development Core Team. (2011) R: A language and environment for statistical computing. R Foundation for Statistical Computing, Vienna, Austria. ISBN 3-900051-07-0. Available: http://www.R-project.org/. Version 2.13.1.

[31] Oksanen J, Blanchet FG, Kindt R, Legendre P, Minchin PR, et al. (2011) vegan: Community Ecology Package. R package version 2.0-2. Available: http://CRAN.R-project.org/packag=egan.

[32] Chao A, Chazdon R, Colwell R, Shen T (2005) A new statistical approach for assessing similarity of species composition with incidence and abundance data. Ecol Lett 8: 148–59.

[33] Chazdon RL, Chao A, Colwell RK, Lin S, Norden N, et al (2011) A novel statistical method for classifying habitat generalists and specialists. Ecology 92: 1332–43. 21797161 10.1890/10-1345.1

[34] Chao A, Lin SY (2011) Program CLAM (Classification Method). Program and User's Guide available: http://purl.oclc.org/clam.

[35] Sturtz S, Ligges U, Gelman A (2005) R2WinBUGS: A Package for Running WinBUGS from R. J Stat Software 12: 1–16 Available: http://cran.r-project.org/web/packages/R2OpenBUGS/index.html..

[36] McCarthy MA (2007) Bayesian Methods for Ecology. Cambridge, UK: Cambridge University Press. p. 88–93.

[37] Lunn D, Spiegelhalter D, Thomas A, Best N (2009) The BUGS project: Evolution, critique and future directions (with discussion). Stat Med 28: 3049–82 Available: http://www.openbugs.info/w/.. 19630097 10.1002/sim.3680

[38] Oliver CD, Larson BC (1990) Forest stand dynamics. New York: McGraw-Hill. 467 p.

[39] Guevara S, Meave J, Morenocasasola P, Laborde J (1992) Floristic composition and structure of vegetation under isolated trees in Neotropical pastures. J Veg Sci 3: 655–64.

[40] Guevara S, Laborde J (1993) Monitoring seed dispersal at isolated standing trees in tropical pastures - consequences for local species availability. Vegetatio 108: 319–38.

[41] Galindo-Gonzalez J, Guevara S, Sosa V (2000) Bat- and bird-generated seed rains at isolated trees in pastures in a tropical rainforest. Conserv Biol 14: 1693–703. 10.1111/j.1523-1739.2000.99072.x 35701928

[42] Guevara S, Laborde J, Sanchez-Rios G (2004) Rain forest regeneration beneath the canopy of fig trees isolated in pastures of Los Tuxtlas, Mexico. Biotropica 36: 99–108.

[43] Harvey CA, Villanueva C, Esquivel H, Gomez R, Ibrahim M, et al (2011) Conservation value of dispersed tree cover threatened by pasture management. For Ecol Manage 261: 1664–74.

[44] Chazdon RL, Vilchez BA, Letcher SG, Wendt A, Sezen UU (2014) Effects of human activities on successional pathways: Case studies from lowland wet forests of northeastern Costa Rica. In press in: Hecht SB, Morrison KD, Padoch C, editors. The social lives of forests: Past, present, and future of woodland resurgence. Chicago: University of Chicago Press.

[45] Holl K (1999) Factors limiting tropical rain forest regeneration in abandoned pasture: Seed rain, seed germination, microclimate, and soil. Biotropica 31: 229–42.

[46] Chazdon R (2003) Tropical forest recovery: Legacies of human impact and natural disturbances. Persp Plant Ecol Evol Syst 6: 51–71.

[47] Laborde J, Guevara S, Sanchez-Rios G (2008) Tree and shrub seed dispersal in pastures: The importance of rainforest trees outside forest fragments. Ecoscience 15: 6–16.

[48] Fischer J, Lindenmayer D (2002) The conservation value of paddock trees for birds in a variegated landscape in southern New South Wales. 2. Paddock trees as stepping stones. Biodivers Conserv 11: 833–49.

[49] de la Peña-Domene M, Martínez-Garza C, Howe HF (2013) Early recruitment dynamics in tropical restoration. Ecol App 23: 1124–34. 10.1890/12-1728.1 23967580

[50] Holl KD, Zahawi RA, Cole RJ, Ostertag R, Cordell S (2011) Planting seedlings in tree islands versus plantations as a large-scale tropical forest restoration strategy. Restor Ecol 19: 470–9.

[51] Harvey CA, Medina A, Merlo Sanchez D, Vilchez S, Hernandez B, et al (2006) Patterns of animal diversity in different forms of tree cover in agricultural landscapes. Ecol Appl 16: 1986–99. 17069389 10.1890/1051-0761(2006)016[1986:poadid]2.0.co;2

[52] Sekercioglu CH, Loarie SR, Brenes FO, Ehrlich PR, Daily GC (2007) Persistence of forest birds in the Costa Rican agricultural countryside. Conserv Biol 21: 482–94. 17391198 10.1111/j.1523-1739.2007.00655.x

[53] Galindo-Gonzalez J, Sosa VJ (2003) Frugivorous bats in isolated trees and riparian vegetation associated with human-made pastures in a fragmented tropical landscape. Southwestern Nat 48: 579–89.

[54] White G, Boshier D, Powell W (2002) Increased pollen flow counteracts fragmentation in a tropical dry forest: An example from *Swietenia humilis* Zuccarini. Proc Natl Acad Sci U S A 99: 2038–42. 11842203 10.1073/pnas.042649999 PMC122315

