# Supplementary material for: Remnant Trees Affect Species Composition but Not Structure of Tropical Second-Growth Forest
Source: PLoS One. 2014 Jan 13;9(1):e83284. doi: 10.1371/journal.pone.0083284 (PMC3890367; doi:10.1371/journal.pone.0083284)
Supplement: File S1 — Supporting Information Document. Resumen, Methods S1, Results S1, and Tables S1-4. (DOCX) [file pone.0083284.s001.docx]

**File S1 - Supporting Information**

**Resumen**

Árboles remanentes que han sido salvados de la tala en bosques tropicales cortados para agricultura o pastoreo, funcionan como núcleos de regeneración forestal luego del abandono de estas actividades. Estudios previos en árboles remanentes han sido llevados a cabo principalmente en sitios de pastoreo activos, o en campos abandonados por 2-3 años, y se han enfocado en la estructura y riqueza de especies del bosque en regeneración, mas no en la composición de especies. Nuestro estudio es uno de los primeros en investigar los efectos de los árboles remanentes en la estructura del bosque, biodiversidad, y composición de especies, 20 años después del abandono. Comparamos la vegetación leñosa alrededor de árboles remanentes individuales con la de parcelas cercanas en los mismos bosques secundarios sin árboles remanentes (“parcelas control”). La estructura del bosque bajo árboles remanentes no fue significativamente diferente a la de las parcelas control. La riqueza y diversidad de especies fueron significativamente más altas alrededor de árboles remanentes. La composición de especies alrededor de árboles remanentes fue significativamente diferente a la de las parcelas control y se asemejó más a la composición de especies en bosques maduros cercanos. La proporción de especialistas de bosque primario y de generalistas alrededor de árboles remanentes fue significativamente mayor que en las parcelas control. Aunque otros estudios han mostrado que árboles remanentes pueden acelerar inicialmente el crecimiento del bosque secundario, nosotros no encontramos que éstos afectan localmente la densidad de tallos, área basal, o densidad de plántulas en etapas posteriores de regeneración. Árboles remanentes, sin embargo, tienen un efecto claro en la diversidad y composición de especies, y grupos ecológicos de la vegetación leñosa cercana, incluso después de 20 años de regeneración del bosque. Para acelerar el retorno de la diversidad y de especies de bosques maduros durante el recrecimiento del bosque en tierras abandonadas, los propietarios deben ser animados a mantener árboles remanentes en los campos agrícolas o de pastoreo.

**Palabras clave**s: árbol remanente; árbol aislado, árbol relicto, bosque tropical, regeneración forestal; sucesión, conservación, regeneración asistida

**Methods S1**

*Additional Study Design*

When choosing central trees for the control plots (“control trees”), we required them to be the top 80^th^ percentile of height and DBH for the area, based on nearby forest survey data of similarly aged secondary forest plots [1]. We chose the top 80^th^ percentile of height and DBH to ensure that the central trees in the control plots were established canopy trees. We used this same forest survey data to determine that trees >75 cm DBH were most likely remnant trees or isolated pasture trees and so should be specifically excluded from our control plots. We also excluded trees >75 cm DBH from remnant plots to ensure that each remnant we chose was >30 m from another remnant or isolated pasture tree. For each remnant and control tree, we measured height to the nearest meter using a Haga Altimeter (Haga Metallwarenfabrik, Nuremberg, Germany). We took three measurements of each tree and averaged them. If the tree had more than one stem at breast height, we measured and recorded the DBH for all stems. Neither height nor DBH of the remnant tree had any effect on surrounding forest structure or species diversity.

*Light Measurements*

In the center of each 1 by 1 m quadrat seedling plot we surveyed, we also took 3 measurements of the red/far red ratio as an index of light availability (Skye SKR 110, Skye Instruments Limited, Llandrindod Wells, UK). We took measurements in rapid succession under overcast to cloudy conditions at a height of 1 m [2].

*Density, Seedling Density, and Basal Area Calculations*

We calculated density and seedling density by summing all trees (≥1 cm DBH) found in a tree plot or number of seedlings, respectively, and dividing by the area surveyed. We calculated basal area per stem (for stems ≥1 cm DBH) by using the standard forestry formula (basal area (m^2^) = π*(DBH/2)^2^) summed over one entire tree plot and divided by the total area in hectares. The 17 *Ficus* spp. trees found in remnant and control plots could not be measured high enough on the trunk to exclude buttresses from the measured DBH. For this reason, the DBH measurements for these trees were multiplied by 0.65, the *Ficus* specific correction suggested by Glenday (2006), to get improved (without buttress) DBH measurements that were then used to calculate basal area [3].

*Additional Statistical Analyses*

We tested whether spatial autocorrelation was present in the data by creating semivariograms in R using the gstat package for basal area, tree density, and species diversity [4]. No evidence of spatial autocorrelation was found.

We performed power analyses to determine the magnitude of detectable differences in basal area, seedling density, tree density, species diversity, or species richness, given our sample sizes. We randomly generated values from a normal distribution with specified means for remnant (10 values) and control plots (20 values). We selected sets of means to determine the smallest possible difference in means for each measured or calculated value that could be detected with our sample sizes.

We tested the sensitivity of our extrapolated species richness, diversity, and evenness analyses to size class by removing all stems ≥5 cm to <10 cm DBH from our richness, diversity, and evenness calculations and re-running the two-way ANOVA using all stems ≥10cm DBH. We did the same for the species composition ANOSIM, the pairwise similarity to old-growth forest, and the specialists and generalists analyses.

**Results S1**

*Power Analyses*

Based on our power analysis and our sample sizes for remnant and control plots, we would have been able to detect a difference of 1 m^2^/ha for basal area, a difference of 1 seedling/m^2^, and a difference of <0.001 trees/m^2^ for density between remnant and control plots. We would have been able to detect a difference of 1 species/individual for extrapolated species richness for all stems ≥5 cm DBH. Our power analysis showed that we would have been able to detect a difference in means of 1.5 for Shannon exponential diversity, and we would have been able to detect a difference in means of 0.5 for species evenness. Note that this while all other measures have enough statistical power to detect small differences in a metric between remnant and control plots, 0.5 is considered a very large difference in terms of species evenness (which is on a scale from 0 to 1).

*Sensitivity Analyses*

Extrapolated species richness, species diversity, and species evenness were not significantly different between remnant and control plots when we used only stems ≥10 cm DBH. This difference in significance is not due to a lack of power as our power analysis showed that we would have been able to detect a difference of 1.5 species/individual for extrapolated species richness and of 1.5 for Shannon exponential diversity for all stems ≥10 cm DBH. ANOSIM showed that species composition between remnant and control plots was different for stems ≥10 cm DBH, as with the ≥5 cm DBH size class. The same was true for our pairwise similarity to old-growth forest analysis. We found more OG Specialists around remnant trees and more SG Specialists in control plots, like in the ≥5 cm DBH size class, but we did not find a significant difference between abundance of Generalists between the two plot types at the ≥10 cm DBH size class.

**References**

1. Morales Salazar MS, Vílchez B, Chazdon R, Ortega M, Ortiz E, *et al.* (2012) Estructura, composición y diversidad vegetal en bosques tropicales del Corredor Biológico Osa, Costa Rica. Recursos Naturales y Ambiente, issue 63 (in press).

2. Capers R and Chazdon R (2004) Rapid assessment of understory light availability in a wet tropical forest. Agric For Meteorol 123:177-85.

3. Glenday J (2006) Carbon storage and emissions offset potential in an east African tropical rainforest. For Ecol Manage 235:72-83.

4. Pebesma EJ (2004) Multivariable geostatistics in S: the gstat package. Computers & Geosciences, 30: 683-691. Available: <http://cran.r-project.org/web/packages/gstat/index.html>

**Table S1. List of remnant and central control trees.**

| Genus | Family | DBH (cm) | 2nd stem DBH (cm) | Ht (m) | Canopy radius (m) | Distance to nearest Old-growth forest (m) | Elev (m) | Dispersal mechanism of focal tree | OC or LR | Remnant, or Control | Classification |
| --- | --- | --- | --- | --- | --- | --- | --- | --- | --- | --- | --- |
| *Apeiba tibourbou* | Malvaceae | 23.6 | --- | 17 | 3.5 | 215 | 43.9 | Animal | OC | Control | SG Specialist |
| *Cordia bicolor* | Boraginaceae | 36.5 | 13.6 | 29 | 5.6 | 60 | 54.6 | Animal | OC | Control | SG Specialist |
| *Cordia bicolor* | Boraginaceae | 29.2 | --- | 27 | 4.0 | 140 | 233.5 | Animal | LR | Control | SG Specialist |
| *Ficus insipida* | Moraceae | 170.5 | 74.7 | 42 | 12.9 | 190 | 43.9 | Animal | OC | Control | SG Specialist |
| *Ficus insipida* | Moraceae | 208.5 | --- | 51 | 16.6 | 315 | 44.8 | Animal | OC | Control | SG Specialist |
| *Ficus insipida* | Moraceae | 156.0 | --- | 38 | 10.5 | 100 | 50.6 | Animal | OC | Control | SG Specialist |
| *Goethalsia meiantha* | Malvaceae | 31.5 | --- | 25 | 3.1 | 50 | 61.6 | Wind | OC | Control | SG Specialist |
| *Hieronyma alchorneoides* | Phyllanthaceae | 29.3 | --- | 30 | 7.7 | 90 | 36.6 | Animal | OC | Control | SG Specialist |
| *Hieronyma alchorneoides* | Phyllanthaceae | 31.5 | --- | 24 | 5.0 | 115 | 246.6 | Animal | LR | Control | SG Specialist |
| *Hieronyma alchorneoides* | Phyllanthaceae | 35.5 | --- | 34 | 4.6 | 100 | 239.3 | Animal | LR | Control | SG Specialist |
| *Inga punctata* | Fabaceae | 24.9 | --- | 24 | 2.4 | 295 | 56.4 | Animal | OC | Control | Rare |
| *Inga multijuga* | Fabaceae | 23.5 | --- | 25 | 3.5 | 150 | 44.2 | Animal | OC | Control | SG Specialist |
| *Pourouma bicolor* | Urticaceae | 25.2 | 3.7 | 19 | 3.7 | 100 | 245.7 | Animal | LR | Control | OG Specialist |
| *Spondias mombin* | Anacardiaceae | 23.5 | --- | 35 | 3.3 | 65 | 45.4 | Animal | OC | Control | SG Specialist |
| *Spondias mombin* | Anacardiaceae | 78.7 | --- | 33 | 9.5 | 60 | 76.2 | Animal | OC | Control | SG Specialist |
| *Spondias mombin* | Anacardiaceae | 85.7 | --- | 40 | 9.9 | 220 | 39.3 | Animal | OC | Control | SG Specialist |
| *Tabebuia chrysantha* | Bignoniaceae | 21.7 | --- | 20 | 2.2 | 55 | 52.7 | Wind | OC | Control | Rare |
| *Virola sebifera* | Myristicaceae | 21.1 | --- | 16 | 4.6 | 95 | 242.0 | Animal | LR | Control | Generalist |
| *Vochysia ferruginea* | Vochysiaceae | 26.0 | --- | 17 | 4.5 | 120 | 263.4 | Wind | LR | Control | SG Specialist |
| *Vochysia ferruginea* | Vochysiaceae | 26.8 | --- | 24 | 3.8 | 50 | 259.1 | Wind | LR | Control | SG Specialist |
| *Caryocar costaricense* | Caryocaraceae | 120.2 | --- | 53 | 11.5 | 70 | 63.4 | Animal, Gravity | OC | Remnant | Rare |
| *Caryocar costaricense* | Caryocaraceae | 201.5 | --- | 55 | 16.0 | 145 | 257.9 | Animal, Gravity | LR | Remnant | Rare |
| *Coccoloba tuerckheimii* | Polygonaceae | 117.0 | 3.9 | 42 | 8.6 | 100 | 257.0 | Animal | LR | Remnant | Rare |
| *Ficus insipida* | Moraceae | 172.5 | --- | 42 | 14.3 | 155 | 283.5 | Animal | LR | Remnant | SG Specialist |
| *Hieronyma alchorneoides* | Phyllanthaceae | 124.5 | --- | 51 | 13.0 | 65 | 54.3 | Animal | OC | Remnant | SG Specialist |
| *Pouteria juruana* | Sapotaceae | 136.5 | --- | 55 | 7.4 | 50 | 258.2 | Animal | LR | Remnant | Rare |
| *Pouteria sp.* | Sapotaceae | 163.5 | --- | 48 | 5.9 | 140 | 271.0 | Animal | LR | Remnant | Rare |
| *Spondias mombin* | Anacardiaceae | 131.4 | --- | 37 | 11.0 | 140 | 247.5 | Animal | LR | Remnant | SG Specialist |
| *Tachigali versicolor* | Fabaceae | 113.0 | --- | 55 | 14.2 | 75 | 58.5 | Animal, Gravity | OC | Remnant | Rare |
| *Terminalia oblonga* | Combretaceae | 163.0 | --- | 53 | 13.3 | 55 | 251.8 | Wind | LR | Remnant | Rare |

We measured DBHs for all trees as high as we could reach on the trunk, but we had no way to measure above the buttresses. Remnant and control tree DBHs were not corrected for buttresses. We measured the distance to nearby old-growth forest from the center of remnant or control tree plots. Average distance from nearest old-growth forest for the 20 control trees was 129.25 m and for the 10 remnant trees was 99.5 m.

**Table S2. Red:far red ratio measurements for all focal trees.**

| **Genus species** | **Family** | **Con/Rem** | **Site** | **Light (R:FR) Mean** | **Light (R:FR) sd** |
| --- | --- | --- | --- | --- | --- |
| *Caryocar costaricense* | Caryocaraceae | Rem | OC | 0.731 | 0.100 |
| *Ficus insipida* | Moraceae | Rem | LR | 0.761 | 0.080 |
| *Pouteria juruana* | Sapotaceae | Rem | LR | 0.658 | 0.076 |
| *Pouteria sp.* | Sapotaceae | Rem | LR | 0.755 | 0.112 |
| *Spondias mombin* | Anacardiaceae | Rem | LR | 0.686 | 0.063 |
| *Tachigali versicolor* | Fabaceae | Rem | OC | 0.709 | 0.070 |
| *Cordia bicolor* | Boraginaceae | Con | OC | 0.823 | 0.103 |
| *Ficus insipida* | Moraceae | Con | OC | 0.743 | 0.143 |
| *Ficus insipida* | Moraceae | Con | OC | 0.495 | 0.093 |
| *Hieronyma alchorneoides* | Phyllanthaceae | Con | OC | 0.746 | 0.091 |
| *Hieronyma alchorneoides* | Phyllanthaceae | Con | LR | 0.596 | 0.064 |
| *Inga multijuga* | Fabaceae | Con | OC | 0.579 | 0.083 |
| *Spondias mombin* | Anacardiaceae | Con | OC | 0.524 | 0.061 |
| *Virola sebifera* | Myristicaceae | Con | LR | 0.752 | 0.030 |
| *Vochysia ferruginea* | Vochysiaceae | Con | LR | 0.651 | 0.078 |
| *Vochysia ferruginea* | Vochysiaceae | Con | LR | 0.618 | 0.053 |

Means are of the red:far red (R:FR) ratio and were obtained by averaging the eight 1 m^2^ plots around the focal tree. Standard deviations are calculated based on sample. The R:FR ratio was not significantly different in remnant plots sampled as compared with control plots sampled, nor was it significantly different between sites. We also found no relationship between seedling density and light.

**Table S3. Top 10 most abundant species around in control or remnant plots.**

|  | **Control** | **%** | **Remnant** | **%** |
| --- | --- | --- | --- | --- |
| 1 | *Apeiba tibourbou* | 8.154 | *Socratea exorrhiza* | 8.403 |
| 2 | *Luehea seemannii* | 5.466 | *Chimarrhis latifolia* | 6.050 |
| 3 | *Lacistema aggregatum* | 5.108 | *Piper spp.* | 5.210 |
| 4 | *Piper spp.* | 5.108 | *Tetrathylacium macrophyllum* | 4.874 |
| 5 | *Spondias mombin* | 4.480 | *Castilla tunu* | 4.202 |
| 6 | *Castilla tunu* | 4.122 | *Croton schiedeanus* | 4.202 |
| 7 | *Cordia bicolor* | 3.853 | *Virola sebifera* | 4.034 |
| 8 | *Simaba cedron* | 3.763 | *Callicarpa acuminata* | 4.034 |
| 9 | *Virola sebifera* | 3.315 | *Alchornea costaricensis* | 2.857 |
| 10 | *Callicarpa acuminata* | 2.957 | *Apeiba tibourbou* | 2.521 |

Numbers given in the % column are percent abundance of that species around the type of focal tree specified.

**Table S4. Species classified as OG Specialist, SG Specialist, or Generalist.**

| **OG Specialist Species** | **SG Specialist Species** | **Generalist Species** |
| --- | --- | --- |
| *Cheiloclinium cognatum* | *Apeiba tibourbou* | *Anaxagorea crassipetala* |
| *Dialium guianense* | *Callicarpa acuminata* | *Brosimum guianense* |
| *Guarea kunthiana* | *Cordia bicolor* | *Brosimum lactescens* |
| *Gustavia brachycarpa* | *Croton schiedeanus* | *Brosimum utile* |
| *Licaria misantlae* | *Cupania rufescens* | *Carapa guianensis* |
| *Pourouma bicolor* | *Ficus insipida* | *Castilla tunu* |
| *Protium ravenii* | *Goethalsia meiantha* | *Chimarrhis latifolia* |
| *Pseudolmedia spuria* | *Guazuma ulmifolia* | *Guatteria amplifolia* |
| *Socratea exorrhiza* | *Hieronyma alchorneoides* | *Inga litoralis* |
| *Tetragastris panamensis* | *Inga multijuga* | *Otoba novogranatensis* |
|  | *Inga thibaudiana* | *Perebea xanthochyma* |
|  | *Lacistema aggregatum* | *Rheedia madruno* |
|  | *Lonchocarpus macrophyllus* | *Simaba cedron* |
|  | *Luehea seemannii* | *Tapirira guianensis* |
|  | *Miconia argentea* | *Tetrathylacium macrophyllum* |
|  | *Palicourea guianensis* | *Vantanea barbourii* |
|  | *Siparuna sessiliflora* | *Virola koschnyi* |
|  | *Spondias mombin* | *Virola sebifera* |
|  | *Trichospermum galeottii* |  |
|  | *Vochysia ferruginea* |  |

We list classified species from the chronosequence survey that were also found in our survey quadrats. The overall abundances of species found in our quadrats but not in the chronosequence survey were low (abundance ≤7), and most species only occurred once in all of our quadrats. In the classification analysis, a species had to have an abundance of at least 10 stems to be classified into a group other than Rare. Therefore, all species found in our survey, but not in the chronosequence survey, were classified as Rare. Most species (122 out of 171) were of sufficiently low abundance to be classified as Rare and are not listed on this table.
