# Supplementary figures and images for: Remnant Trees Affect Species Composition but Not Structure of Tropical Second-Growth Forest
Source: PLoS One. 2014 Jan 13;9(1):e83284. doi: 10.1371/journal.pone.0083284 (PMC3890367; doi:10.1371/journal.pone.0083284)

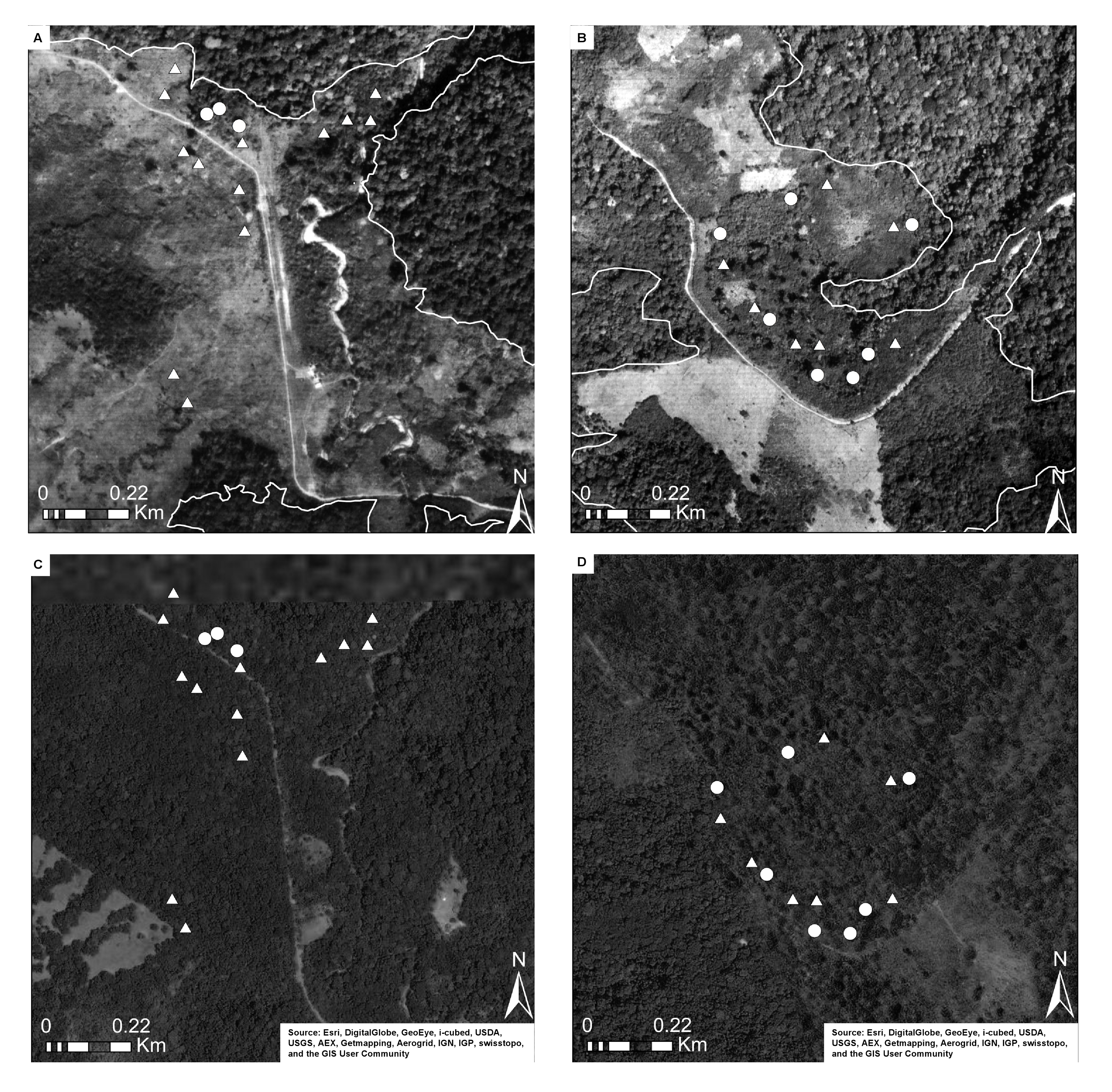

Supplement: Figure S1 — Land cover change and tree plot locations at Osa Conservation and Lapa Ríos. Remnant trees (white dots), and control trees (central trees in control plots; white triangles) on A) and C) Osa Conservation land and B) and D) Lapa Ríos Ecolodge and Wildlife Reserve land in A) and B) 1976 and C) and D) 2013. The white lines on the 1976 aerial photographs indicate the boundary of old-growth forest at greatest known level of clearing (not necessarily 1976). The images were processed in ArcMap 10.1. Photographs from 1976 are courtesy of Instituto Geográfico Nacional (San Jose, Costa Rica). Current (2013) satellite imagery is from ESRI basemap World_Imagery layer; satellite imagery sources are cited in the bottom right of both of the images. (TIF) [file pone.0083284.s002.tif]
